# Oxygen-Carrying Micro/Nanobubbles: Composition, Synthesis Techniques and Potential Prospects in Photo-Triggered Theranostics

**DOI:** 10.3390/molecules23092210

**Published:** 2018-08-31

**Authors:** Muhammad Saad Khan, Jangsun Hwang, Kyungwoo Lee, Yonghyun Choi, Kyobum Kim, Hyung-Jun Koo, Jong Wook Hong, Jonghoon Choi

**Affiliations:** 1 School of Integrative Engineering, Chung-Ang University, Seoul 06974, Korea; saad.engr@gmail.com (M.S.K.); isnickawesome@gmail.com (J.H.); orztapa@gmail.com (K.L.); dydgus5057@gmail.com (Y.C.); 2 Division of Bioengineering, Incheon National University, Incheon 22012, Korea; kyobum.kim@inu.ac.kr; 3 Department of Chemical and Biomolecular Engineering, Seoul National University of Science and Technology, Seoul 01811, Korea; hjkoo@seoultech.ac.kr; 4 Department of Bionano Technology, Hanyang University, Seoul 04763, Korea; 5 Department of Bionano Engingeering, Hanyang University, Ansan 15588, Korea

**Keywords:** microbubbles, nanobubbles, photoacoustic imaging, ultrasonic imaging, reactive oxygen species (ROS), oxygen delivery

## Abstract

Microbubbles and nanobubbles (MNBs) can be prepared using various shells, such as phospholipids, polymers, proteins, and surfactants. MNBs contain gas cores due to which they are echogenic and can be used as contrast agents for ultrasonic and photoacoustic imaging. These bubbles can be engineered in various sizes as vehicles for gas and drug delivery applications with novel properties and flexible structures. Hypoxic areas in tumors develop owing to an imbalance of oxygen supply and demand. In tumors, hypoxic regions have shown more resistance to chemotherapy, radiotherapy, and photodynamic therapies. The efficacy of photodynamic therapy depends on the effective accumulation of photosensitizer drug in tumors and the availability of oxygen in the tumor to generate reactive oxygen species. MNBs have been shown to reverse hypoxic conditions, degradation of hypoxia inducible factor 1α protein, and increase tissue oxygen levels. This review summarizes the synthesis methods and shell compositions of micro/nanobubbles and methods deployed for oxygen delivery. Methods of functionalization of MNBs, their ability to deliver oxygen and drugs, incorporation of photosensitizers and potential application of photo-triggered theranostics, have also been discussed.

## 1. Introduction

The lack of oxygen, also known as hypoxia, is a common characteristic of solid tumors, which results in reduced therapeutic response and malignant progression [1,2,3]. Hypoxic areas in tumors develop owing to an imbalance of oxygen supply and demand. Tumor progression owing to rapid cellular growth and alterations in the microenvironment of tumor cells leads to an inadequate supply of oxygen, resulting in hypoxic conditions [1,2,3,4]. The oxygen level is not the same for all tissues. The physiological range of oxygen is 4–8%, which translates to the oxygen partial pressure of 40–60 mmHg, instead of the atmospheric level of 21% oxygen, while cellular hypoxia is defined as an oxygen level of 1–5% for most tissues, which is in the order of oxygen partial pressure of 10 mmHg [4,5]. Hypoxic cells are more resistant to radiotherapy and chemotherapy than normoxic cells [2,4,5]. Increasing oxygen partial pressure at the cellular level could increase the sensitivity of tumors to radiation therapy by increasing the oxygen enhancement ratio (OER) [5]. Tumor cells under hypoxic conditions slowly proliferate, thereby reducing the effectiveness of the chemotherapeutic agents [4]. Photodynamic therapy (PDT) is another treatment mechanism that is oxygen-dependent for the generation of reactive oxygen species (ROS), and the increased oxygen level enhances the effectiveness of PDT [6,7].

Hypoxemia or low blood oxygen level occurs in patients suffering from severe lung injury, airway obstruction, reduced cardiac output, or any critical illness, and hypoxemia is responsible for an increased mortality rate [2,8,9]. Hypoxemia may cause cardiac arrest or organ damage and neurological complexities owing to low oxygen supply to the brain [2,10]. Severely hypoxemic patients are treated with inspired oxygen or mechanical ventilation, and if these measures are delayed or insufficient, they may lead to organ injury or death [8]. Treating an airway fracture or a lung collapse with mechanical ventilation is not sufficient [8,11].

Several therapeutic methods have been employed by researchers to increase oxygen tension. Increasing the red blood cell count to improve pO_2_ levels in tumors has not shown significant results [2]. Hemolysis may occur if free oxygen gas bubbles are directly injected into human blood [12]. Therapeutic interventions to increase oxygen tensions, such as carbogen inhalation and hyperbaric oxygenation, have clinical and logistic limitations and, therefore, there is a need for an oxygen source that is practical, cost-effective, biocompatible, biodegradable, non-toxic, and can deliver oxygen in a higher concentration to reverse the hypoxic and hypoxemic conditions [5,8,13,14,15,16].

Micro/nanobubbles (MNBs) are spherical vesicles consisting of a shell and core, and they have been used as ultrasound contrast agents for several decades in the field of medicine [10,17,18,19,20]. The term microbubble is commonly used in reference to ultrasound contrast agents owing to their micrometer size [21,22]. Microbubble-based contrast agents have also been used for a photoacoustic imaging technique, which is a non-invasive real-time molecular imaging technique based on the optical absorption of tissues [23,24,25]. Nanobubbles have been investigated for diagnostic and therapeutic purposes owing to their nanometer size, for enhancing cellular penetration of these bubbles [22,26,27,28,29,30,31]. Tumors exhibit leaky vasculature and various researchers have investigated the effect of the enhanced permeability and retention (EPR) effect, which is the ability of tumors to accumulate particles in the size range of 380–780 nm. Yin et al. demonstrated that nanobubbles exhibited similar echogenic properties as microbubbles when high frequencies of ultrasound were used and nanobubbles were retained in tumors for longer periods as compared to microbubbles [32]. Various other researchers have also investigated nanobubbles as ultrasound contrast agents for tumor imaging and drug/gene delivery applications [2,31,33,34,35]. Compared to nanosized liposomes, which contain a lipid bilayer membrane and hydrophilic aqueous core, nanobubbles have monolayer shells encapsulating a hydrophobic gas core, making them feasible for gas delivery applications [20,26,36,37]. Both microsized and nanosized bubbles have been used for oxygen delivery [15]. Therefore, in this review, the term micro/nanobubbles (MNBs) was used to address the similarity of the properties of these vesicles in relation to the oxygen supply and their applications. A higher surface contact area, smaller size, polydisperse size distribution, higher payload, higher cellular uptake, and an efficient gas delivery mechanism are promising aspects of MNBs, and these attributes make MNBs suitable for gas and drug delivery applications [12,38,39,40,41,42,43]. 

The term theranostics refers to the combination of therapeutic and diagnostic modalities [44]. Integration of therapeutic and diagnostic technologies are aimed at improved and personalized treatment, taking advantage of nanomedicine and nanotechnology. Photo-based stimuli for activation of imaging and therapeutic modalities are gaining popularity because of their reduced side effects and dual activation mechanism [45,46].

This review provides a general overview of MNBs used as a source of oxygen to reverse hypoxia and hypoxemia during the last two decades. The general characteristics and composition of the MNBs, synthetic techniques, methods deployed for oxygen delivery, shortcomings, and prospects in photo-triggered theranostics related to photodynamic therapy and photoacoustic imaging are discussed. 

## 2. Characteristics and Compositions of Micro/Nanobubbles (MNBs)

MNBs are echogenic particles, and they respond to applied ultrasonic fields. Echogenicity is an inherent property of MNBs owing to the encapsulated gas inside the shell, causing a difference in the acoustic impendences of the shell and gas due to which ultrasound backscatter is enhanced [30,47]. The most promising property of MNBs is efficient gas solubility inside the shell [48]. The core usually consists of a medical gas, while the shell is composed of biomolecules, such as lipids, proteins, polymers, and surfactants [12,49,50]. MNBs oscillate when acoustic waves are applied owing to the difference in the density of the gas and surrounding aqueous solution [51]. At low acoustic pressures, MNBs oscillate persistently, a phenomenon known as stable cavitation that enhances the diffusion of the core gas out of the bubble [2,36,52]. MNBs exhibit violent expansion and contraction at higher amplitudes of applied acoustic fields, which results in the violent collapse and fragmentation of the microbubble, known as inertial cavitation [2,51,53].

**Figure 1:**
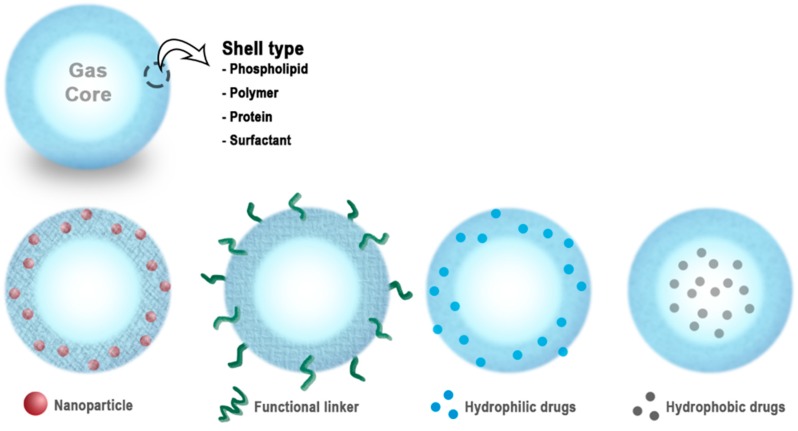
Schematic of micro/nanobubbles (MNBs), and their functionalization. This figure has been adapted from various studies [22,47,49,55,56].

The schematic diagram in Figure 1 shows general structures of MNBs. The shell is a monolayer of a hydrophilic or amphiphilic biomaterial that encapsulates the core gas [54]. This simple structure enables the MNBs to be used in a variety of medical applications, including molecular imaging, gas delivery, drug delivery, and gene therapy. The shells of the MNBs can be engineered for various applications. MNBs can be bio-conjugated with various types of drugs or proteins/DNA for targeted delivery [55]. 

For diagnostic and therapeutic applications, the characteristics of MNBs, such as size, stability, shell type, biocompatibility, and core gas, are important parameters, and they must be tailored according to the required applications [17]. These factors are summarized below. 

### 2.1. Size and Stability

Size is a limitation of MNBs, which must be small enough to pass through blood capillaries. Even though larger bubbles are more echogenic owing to the high backscatter of ultrasound, they can block systemic capillaries. Consequently, MNBs in the range of 0.1 to 20 μm have been used in most of the applications [47]. The size uniformity and productivity of MNBs depend upon the composition and synthetic techniques employed [57].

The stability in the MNBs is governed by the pressure difference between the interior and exterior of the shell, also known as the Laplace pressure. The pressure difference is given by the following equation [22,30,57,58]:

(1) $$\Delta P=Pin-Pout=2\frac{\delta }{r}$$

where *δ* is the interfacial tension and *r* is the radius of the bubble. This equation indicates that the Laplace pressure increases when the size of the bubble decreases, and as the gas diffuses out of the core, the Laplace Pressure increases substantially to rupture the bubble. Therefore, to stabilize MNBs, various formulations have been evaluated to decrease the surface tension and increase their stability [57]. Surfactants play an important role in stabilizing MNBs, as they reduce the interfacial tension *δ* and thus reduce the Laplace pressure at smaller radii of the MNBs [30,41,43]. The stability of the MNBs has been studied using various surfactants, proteins, solid particles, and PEGylated lipids to avoid disproportionation and coalescence [57].

### 2.2. Shell Types

Free air bubbles that are not stabilized by any type of shell dissolve rapidly [59,60]. The stability and biocompatibility of the MNBs and release of the core gas depends on the type of the shells and the core gas used [39,57]. The shell forms a protective layer around the gas to provide stability and protection from endogenous scavengers, and it reduces the rate of diffusion of the core gas into the surrounding media [2,47,49,54]. The shell composition determines the stiffness, elasticity, gas exchange, half-life, resistance against the applied ultrasonic pressure, and the ease in excretion of the MNBs from the body [30,47]. If the shells are soft, they will break easily, while hard shells will not be able to oscillate in ultrasonic fields [30]. Shell composition is an important factor in the loading of drugs and genes. Therefore, it is important to choose appropriate shell materials for diverse applications of MNBs with various thicknesses, stiffnesses, charges, and functional groups [54].

MNBs exhibit shelf life ranging from a few weeks to several months depending upon the type of shell materials. Swanson et al. reported protein-shelled microbubbles losing almost half the oxygen gas in a 12-day storage period [40]. Kheir et al. reported that lipid-shelled oxygen bubbles retained 80% of the gas over a two-week observation period [10]. Polymer shells have been reported to have a shelf life of approximately six months. To increase the shelf life, various techniques have been adopted. The in vivo half-life of MNBs ranges from a few seconds to several hours, again depending upon the shell composition. The main properties of various shell types have been summarized below.

#### 2.2.1. Lipid Shells

Lipid-based nanoparticles and microparticles are preferred for medical applications because of their biocompatibility and biodegradability. Lipids form flexible shells that are approximately 3 nm in thickness [30]. These lipid shells allow diffusion of gas through the shells, and they show improved resonance under acoustic pressures. Phospholipids are frequently used in lipid-shelled MNBs, as they are amphiphilic with a hydrophilic head and hydrophobic tails [18,61]. Phospholipids can self-assemble into monolayers at the gas–water interface, and the gas or hydrophobic drugs can be encapsulated inside these lipids shells [10,62]. Phospholipids can be used to synthesize MNBs through various techniques, including sonication and mechanical agitation. Lipids are highly cohesive, which provide solid-like characteristics to these MNBs. The rigidity of the lipid shells depends on the type of lipids used. Longer hydrocarbon chains impart more rigidity in the shells [30]. Lipid-coated MNBs can be functionalized with an appropriate concentration of emulsifiers. They are stable at the nanosize scale, showing the potential for a variety of applications in drug delivery [22,49,62].

Various combinations of base phospholipids, such as 1,2-distearoyl-sn-glycero-3-phosphocholine (DSPC) and 1,2-dipalmitoyl-sn-glycero-3-phosphocholine (DPPC) have been used by researchers in combination with various surfactants and emulsifiers, including polyethylene glycol modified (PEGylated) lipids [20,63]. Polyethylene glycol (PEG) is highly hydrophilic with low toxicity, and it is used to improve stability and surface modification and avoid coalescence [11,64,65]. PEG incorporated into the lipid increases the half-life, reduces immunogenic response, prolongs circulation, and decreases plasma clearance [61,64,65,66,67]. Cholesterol has been used to strengthen the lipid monolayer and decrease diffusion rate [12]. Adding PEG-biotin to the lipid shell helps in attaching avidin conjugated dye, such as fluorescein isothiocyanate (FITC), which will produce fluorescent MNBs. Various drugs can also be attached to the lipid shells if they have some affinity with the lipids [10,39,49,62,68]. 

#### 2.2.2. Protein Shells

Protein-shelled MNBs are favorable for their stability, biocompatibility, biodegradability, amphipathic nature, and longer half-life [18,40,62,69]. Protein-shelled MNBs are synthesized by heating the protein solution to the denaturing point and emulsifying them. The denatured protein forms a thin monolayer shell across the desired gas [30,40,70]. The protein shells are rigid, and diffusion across them is limited. This rigidity might be explained by disulfide bonding that occurs between the thiol groups of cysteine [2,62]. Albumin-shelled MNBs have been used for an ultrasound contrast agent. Commercial products, such as Albunex, are approved by the Food and Drug Administration (FDA) and used for commercial applications. They contain bubbles with a diameter of 1–15 µm and a shell thickness of 15 nm. PEGylation has also enhanced the stability and shelf life of protein-shelled MNBs [70]. Several other bioactive proteins, such as avidin, have also been used for producing shells of MNBs with diverse functionality [62]. Thiol-rich proteins, such as human serum albumin, have also been identified as suitable for cross-linking to form shells of MNBs [30]. Protein-shelled oxygen microbubbles have been used for oxygen delivery to an oxygen-depleted saline solution [40]. 

#### 2.2.3. Polymer Shells

Polymer shell bubbles are thicker than lipid and protein shells, sometimes in the range of 150–200 nm, thereby enabling polymeric bubbles to have a higher drug-loading capacity for hydrophobic and hydrophilic drugs [47,60,62]. Polymeric shell bubbles are more resistant to compression and expansion when ultrasound fields are applied. They exhibit low oscillations at lower acoustic pressures and crack or defect when higher pressures are applied, allowing the gas or drug to diffuse out [47]. Biodegradable polymers have also been used by researchers for oxygen delivery. Chitosan, poly(lactic acid) (PLA), poly(vinyl alcohol) (PVA), poly(glycolic acid) (PGA), and poly(lactic-co-glycolic acid) (PLGA) have been used to synthesize polymeric shell MNBs because of their stability, biocompatibility, reproducibility, biodegradability, and purity [13,28,30,71]. PLGA with a higher fraction of glycolic acid has a longer shelf life [30]. Chitosan-shelled MNBs can deliver oxygen without the application of ultrasound, owing to their biodegradability and thin shell formulation [13]. Dextran-coated MNBs incorporating PVA in the shells have been used for oxygen delivery in hypoxic conditions [72]. MNBs having polymeric surfaces can also be PEGylated to improve biocompatibility and reduce immunogenicity [28]. Polymerization techniques have also been employed to develop nanocarriers for PDT. For example, a linear block copolymer (BCP) has the ability to form a hydrophobic core to incorporate a photosensitizer drug and a hydrophilic headgroup [6]. Polymer-shelled MNBs have also been preferred for molecular imaging due to higher stability and strong stimulated acoustic signals [73].

### 2.3. Fate/Excretion/Biocompatibility/Biodegradation Issues

Microbubbles have been in commercial use as FDA-approved ultrasound contrast agents for several decades [62]. When injected into the bloodstream, MNBs are captured by the cells through endocytosis processes [74]. Various researchers have tested the biocompatibility of MNB formulations and have shown that they are biocompatible [47]. Lipids and proteins are biomolecules, and they are metabolized by the body through normal mechanisms. Among polymers, biopolymers are more favorable for the synthesis of MNBs given their ability to biodegrade. PEGylation improves immunogenic properties [59]. Surfactants have been tested to avoid the coalescence of MNBs to maintain smaller sizes and avoid blockage of capillaries. MNBs have been shown to be non-hemolytic [13].

### 2.4. Core Gas

MNBs can be injected intravenously. For a contrast agent, sustainable MNBs are required to provide contrast for a longer period. A low- or medium-intensity ultrasound is applied for imaging purposes to create bubbles that are in a stable cavitation phase. Various researchers have studied pure oxygen delivery through MNBs. Oxygen MNBs dissolve quickly and release the core gas without requiring ultrasound, especially for lipid shells [12]. Improved stability is obtained using a hydrophobic gas with high molecular weight inside the MNBs, which has low solubility [63,75]. Therefore, for a longer duration of circulation, non-polar heavy gases, such as perfluorocarbons (PFC) and sulfur hexafluoride (SF_6_), have been used in combination with oxygen gas to increase the stability and longevity of MNBs [22,72,76]. Oxygen is a water-soluble gas, while PFCs are hydrophobic. PFCs dissolve oxygen and provide stability to the MNBs against the external Laplace pressure, while oxygen is exchanged with external gases across the concentration gradient [76]. Mixed-gas oxygen and C_3_F_8_ have been used for the stable delivery of oxygen [16]. A mixture of 95% O_2_ and 5% PFC was also used for the stable release of oxygen [5]. Gases other than oxygen, such as NO, have also been delivered through MNBs [71].

### 2.5. Characterization Techniques for MNBs

Various characterization techniques have been applied to characterize MNBs. Optical and fluorescence microscopy can reveal micron-sized bubbles, while dynamic light scattering (DLS) and other optical particle counters can be used to determine the size of the nanobubbles [8,10,13,74]. Scanning electron microscopy (SEM) and transmission electron microscopy (TEM) techniques have been used to visualize MNBs [12]. The methods to measure dissolved oxygen include electrochemical sensing, fiber optic-based sensing, and fluorescence quenching [77]. Electrochemical sensing has also been applied to measure the dissolved oxygen content in a solvent subsequent to the injection of oxygen containing MNBs in a deoxygenated solvent [13]. Fluorescence quenching uses a live cell-imaging technique when cells are under hypoxia, and it can be used to evaluate the performance of MNBs to reverse hypoxia [20,78]. Fiber-optic based sensors have also been employed to measure a change in oxygen concentration owing to the MNBs [5]. Oxygen measurements in 3D cell cultures were conducted using needle-type oxygen microsensors based on fiber optics [79]. Researchers have also estimated the content of oxygen in MNBs by calculations of density, particle size, and the number of particles [11]. Degradation of HIF-1α in in vivo models has also been used as a method to reverse the hypoxic condition after injection of MNBs. To test the applications of MNBs for oxygen delivery, researchers have tested the mechanisms by oxygen delivery to deoxygenated water, cells under hypoxic conditions, and animals kept in hypoxemia models. 

## 3. Synthetic Techniques for MNBs

MNBs are synthesized by various methods. Popular methods include sonication, agitation, microfluidic devices, and laser ablation [48].

### 3.1. Sonication

Sonication is a popular method to synthesize MNBs in a single step [57]. This method is applied to synthesize MNBs of various types of shell coatings including lipids, polymers, proteins and surfactants. When ultrasound is applied in a medium, the compressions and rarefactions produce high-pressure and low-pressure zones in the fluid. In addition, if there are surfactants or coating materials present in the medium, the ultrasonic pressure may destroy them, thereby causing them to take new forms, thus stabilizing the gas–liquid interface by the formation of MNBs [22,57]. The process of cavitation and bubble formation is not fully understood. Sonication is a stochastic method and, therefore, generates MNBs of random sizes. However, the size distribution can be controlled using sonication parameters, such as frequency, pulse duration, and power [17]. Researchers have used sonication in the range of 100 to 200 W for approximately 1 to 5 min in a pulsed mode to create MNBs [20,53]. 

Commercial sonicators like bath-tub and tip sonicators are also available for synthesizing MNBs. The limitation of the sonication method is the productivity and yield of the MNBs. This is because only a limited amount of solution can be sonicated within a given time. Therefore, researchers have investigated other methods for a high yield [12]. 

### 3.2. Ink-Jet Method

Microbubble synthesis has been performed using an ink-jet method, in which a polymer solution is forced through a piezo-driven ink-jet nozzle of a desirable size, depending on the application. The piezoelectric crystals create pulses in the solution and the bubbles that are formed are removed from the nozzle [17,57]. A similar method has also been applied to generate ultrafine oxygen nanobubbles from pure water and an oxygen supply by utilizing a high-pressure flow through the nozzle [80].

### 3.3. Microfluidic Techniques

Microfluidic devices have the ability to synthesize MNBs with controlled size distributions. Flow rate, pressure, viscosity of the liquid solution, and the orifice size of the device can be controlled to determine the size and distribution of the MNBs [22,57]. To the two main methods for the fabrication of microfluidic devices are as follows: (1) Soft lithography techniques to produce flow focusing units; and (2) mechanically assembled units from capillaries assembled in a polymeric block. The gas and liquid flows into a T-junction in both cases The MNBs are then generated in the T-junction depending on the size of orifice and other parameters of the device being used [17].

### 3.4. Laser Ablation Method

The laser ablation method is also a stochastic method that can generate MNBs. An excimer laser of a particular wavelength can be focused onto aluminum oxide particles in water, which then forms oxidized nanoparticles. During the process, bubbles will also be produced at the solid–liquid interface. The bubbles/interface are stabilized by the aluminum oxide nanoclusters [57].

### 3.5. Agitation Method 

MNBs, especially those having lipid shells, can be produced by agitating the liquid solution at several thousand oscillations per minute in a shaker. This will produce bubbles with a random size distribution [22,81]. To encapsulate a given gas in an MNB, the container is filled with the desired coating material in the liquid phase and the gas is perfused from the top and then the container is mechanically agitated so that the shell material encapsulates the desired gas [82]. Mechanical agitation is a promising method to produce MNBs on an industrial scale [29]. 

### 3.6. Emulsification Method 

This method is usually applied to synthesize polymer shell MNBs [22]. In this process, water is formed in an oil emulsion with a carrier polymer, and this emulsion is further emulsified in a large volume of water. The solvent is evaporated or extracted to obtain a solid polymer shell, and lyophilized shells are refilled with core gas, such as PFCs [28]. A high-shear emulsification method has been used to synthesize MNBs with a broader size range [57]. A membrane emulsification method can be used to generate MNBs with a narrow size distribution. A porous membrane is used for this purpose. Gas bubbles permeate and disperse into a continuous phase flowing along the membrane surface. Emulsifiers are added to prevent coalescence [57].

## 4. Reversal of Hypoxia by Oxygenated MNBs 

MNBs are injectable into the circulation system and can create supersaturated suspensions. Therefore, they have been used for increasing the oxygen content and reversing hypoxia [40]. Overcoming with the effects of hypoxia has been studied by researchers improving the efficacy of photodynamic therapy, chemotherapy and radiotherapy [16]. Hypoxic conditions lead to overexpression and stabilization of the hypoxia inducible factor-1α (HIF-1α) protein which has been reported as major cause of higher resistance in therapeutic interventions. HIF-1α has been associated with an increase in anaerobic metabolism, upregulation of genes involved in apoptosis, angiogenesis and pH regulation. These factors are involved in higher tumor resistance and poor prognosis [50,77,83,84,85,86]. Ji et al. reported the effect of overexpression of HIF-1α on cellular resistance towards photodynamic therapy in Het-1A cells and concluded that overexpression of HIF-1α reduces the efficacy of photodynamic therapy due to the induction of angiogenesis and increase in cellular resistance [87]. An adequate supply of oxygen to a 3D scaffold to avoid hypoxic conditions is also a concern in bone tissue engineering [79,88]. Various researchers have targeted inhibition/silencing of HIF-1α to improve tumor treatment [20,89]. 

Oxygenated MNBs have been investigated by several investigators to reverse the hypoxic state and provide more oxygen to cancerous tissue than the tissue level partial pressure [15,26,27,53,72]. Cavalli et al. reported the use of oxygen nanobubbles for delivery of oxygen to hypoxic solutions [72]. Owen et al. demonstrated that oxygen nanobubbles can be delivered orally to reduce hypoxia in tumor microenvironments [15]. McEwan et al. demonstrated improvement in cytotoxic effects of drugs in sonodymanic therapy with oxygen-loaded microbubbles [53]. Bhandari et al. also showed reversal of hypoxia using oxygen nanobubbles to weaken hypoxia-driven pathways [27]. Various researchers have demonstrated that the degradation of HIF-1α can be achieved by providing adequate oxygen through the MNBs, which may increase treatment efficacy [20,90]. Increasing oxygen partial pressure at the cellular level may increase the efficacy of drugs and radiation. The OER is a key factor in the sensitization of tumor cells to radiotherapy. Improving the OER has been proven to improve the effectiveness of radiotherapy [5]. Therefore, improving oxygen levels in tumor tissues and downregulation of HIF-1α has been correlated with improved treatment in chemotherapy, photodynamic therapy and radiotherapy.

MNBs with oxygen as the core gas can be used for the reversal of hypoxemia in the blood [12]. Under normal conditions, red blood cells are required for supplying oxygen to the entire body. The body cannot receive more oxygen than the red blood cells supply through natural respiration mechanism. If blood loss or the loss of function of the lungs were to occur, then sufficient oxygen cannot be supplied to the body. Injecting oxygen containing MNBs can provide sufficient oxygen to the body for a period of 20–30 min, which is vital for patients to reach the hospital and receive emergency treatment. It has been demonstrated that MNBs can be supersaturated with oxygen and the gas can be effectively delivered to degassed water, venous blood or hypoxic tissues without cytotoxic effects [10,12,14,20,22]. Thus, oxygen filled MNBs can prove to be sufficient for the reversal of hypoxia and improving blood oxygenation.

**Figure 2:**
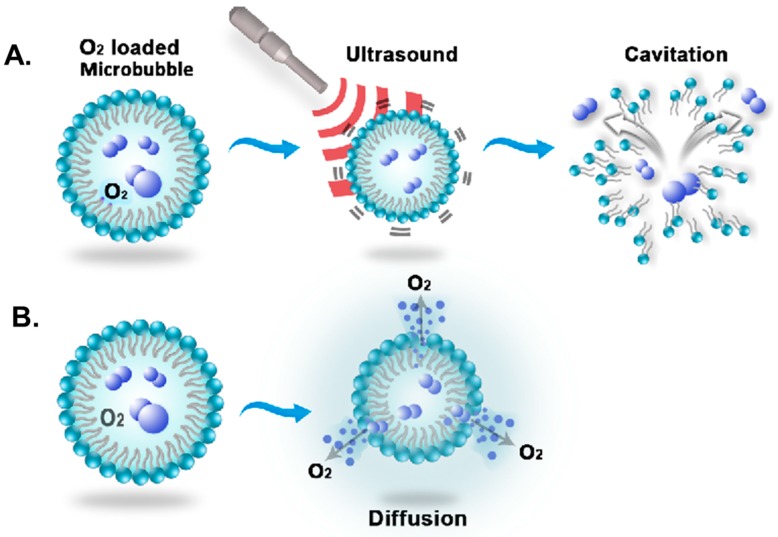
Oxygen delivery mechanism of MNBs: (**A**) MNB disruption using ultrasound and (**B**) diffusion of oxygen across the concentration gradient.

Two main methods for delivering gas via MNBs are shown in Figure 2. One method is to inject MNBs intravenously; subsequently, the MNBs are broken by applying high-intensity ultrasound. High-intensity ultrasound creates zones of high and low pressure along its propagating wave owing to the bubbles resonating and rupturing, thereby releasing the core gas [2,5]. This phenomenon is known as inertial cavitation, and it also creates pressure inside the tissue that temporarily increases the tissue permeability. Hence, large drug molecules can penetrate the tissues and cells [30,62]. Ultrasound mediation also help in controlled release of the drug/genes at specific targets [62]. A second method is to allow diffusion of gas along the concentration gradient. Most of the shells of MNBs are permeable to gas, especially soluble gases like oxygen, which can diffuse out relatively easily. It has been demonstrated that lipid-shelled microbubbles easily allow diffusion across the concentration gradient [12]. This diffusion is a spontaneous process. However, the bubbles may not break completely, and the core gas may be released slowly for polymeric shells, as reported by Cavalli et al in case of Dextran nanobubbles [72]. Drug delivery by the MNBs is also done by similar mechanisms. Ultrasound can be used to break the shell and release the drug immediately, or the the bubbles will break due to increasing external Laplace pressure after the core gas diffuses out, shrinking the size of the bubble. MNBs can be taken up by the cells through endocytosis. If shells are made up of lipids or proteins, they are metabolized by natural cellular processes. In the case of polymers, biodegradable polymers are preferred for drug-delivery applications [22,30,63,75,90]. Both methods for oxygen delivery have been depicted below in Figure 2.

Table 1 lists the shell types, composition, oxygen delivery methods, synthesis techniques, and general properties of MNBs. This table is limited to MNBs that are used only for oxygen delivery applications.

## 5. Functionalization of MNBs with Drugs, Genes, and Targeting Ligands 

The main purpose of loading MNBs with drugs and genes is to minimize the side effects associated with these bioactive substances, along with improving therapeutic efficacy by lowering the effective dosage and minimizing the interference while reaching the target site [52]. MNBs can be used for both passive and active targeting. Passive targeting refers to the tendency of the MNBs to accumulate at tumor sites owing to the leaky vasculature. The effect is also known as enhanced permeability and retention (EPR). Tumor vasculature is irregular and contains large pores within the range of 300–700 nm. MNBs in this size range have the benefit of EPR [2]. Physical properties of MNBs like elasticity, porosity, surface charge, size and shell composition and their interaction with tumor microenvironment play a great role in the EPR effect [92,93]. Higher EPR would translate into higher uptake, better biodistribution and more bioavailability of the drug, resulting in more effectiveness and better treatment. A higher cellular uptake of MNBs owing to endocytosis makes them suitable for drug delivery applications. 

Surface modification of MNBs is required for active targeting by attaching some targeting ligands. This can be achieved by attaching bioactive molecules to the shell of the MNBs [94]. Targeting MNBs can be created by incorporating targeting ligands, such as biomarkers, antibodies, polysaccharides, or other active biomolecules in MNBs [52,74]. Inflammation, vascular clots, endothelial receptors, and specific antigens can be potential targets for such MNBs [52]. Researchers have used tumor cell folate receptors as targets by incorporating folic acid PEG-conjugates in liposomes [95]. The ability to successfully target and capacity to deliver specific drug/gas depends upon the structure and compositions of MNBs [93]. Functionalized MNBs have been used for contrast enhancement in targeted fields for ultrasonic molecular imaging. In this method, targeted MNBs are injected and allowed to adhere to the target tissue, determining the contrast difference that resulted due to accumulation of functionalized MNBs in the target tissue [19,38,62]. Stimuli-responsive MNBs can be synthesized with tunable functional properties. The stimuli responsiveness of these MNBs will originate from the building blocks used in their shell structure [57]. Three methods can be applied to the functionalization of the MNBs. First, MNBs can be synthesized with biomolecules/bioactive substances incorporated in the shell or inside the core of the MNBs. Hydrophilic and amphiphilic biomolecules can be incorporated in the shell while hydrophobic drugs can be loaded in the core of MNBs. The drug-loading capacity is dependent on the type of the shell employed. Thin phospholipid shells are more echogenic and favorable for hydrophilic molecules while thick polymeric shells are preferred for hydrophobic drug loading in the core [2,17,18,55]. Second, covalent and non-covalent techniques can be applied for functionalization of the MNBs by attaching targeting ligands to the protein, polymer, or lipid-based shells. This method is favorable for hydrophilic drugs for targeted delivery [30,81,96]. Biotin-avidin linkages can be incorporated in MNBs for linking antibodies and proteins [2]. MNB shells can be made cationic to apply electrostatic interactions for gene delivery. This method facilitates the gene therapy and various researchers have aimed at using MNBs to enhance targeted gene delivery [17,47]. Finally, MNBs can be co-administered with bioactive substances, using high-intensity ultrasound to enhance cell permeability for a higher uptake of the bioactive molecules [17,18,97]. 

## 6. Potential Applications of Photoacoustic Imaging and Photodynamic Therapy

Photoacoustic imaging is a real-time non-invasive technique for molecular imaging that applies the optical properties of tissues with an optical contrast agent [24]. Photoacoustic imaging is based on the thermoelastic expansion of the tissue due to the absorption of optical energy of applied laser light in the presence of optical contrast agents. Due to thermoelastic expansion, a pressure wave is generated by the tissue which is collected by an ultrasound transducer [98]. Photoacoustic imaging combines the benefits of optical imaging and ultrasound and provides the ability to visualize a tumor located in a tissue [99]. Additionally, photoacoustic imaging can be used to measure oxygen saturation in a tumor microenvironment by measuring oxygenated and deoxygenated hemoglobin [100]. Photoacoustic imaging requires three components: A laser source for irradiation, a photosensitizer drug at the target site, and an ultrasound transducer. The laser source activates the photosensitizer drug which converts the light energy into heat and the ultrasound transducer is used to collect the resulting acoustic signal generated by the photoacoustic effect in the tissues [98,99]. Cancer detection and tumor growth using the photoacoustic imaging technique has been investigated by various researchers [99]. A photosensitizer drug may require a suitable carrier to be effectively delivered to the target tissue. A contrast agent that can respond to light (photosensitizer) and ultrasound simultaneously, would enhance the dual-imaging capabilities, providing anatomic, functional, and molecular imaging signals [24]. Especially for photoacoustic imaging, plasmonic nanoparticles and near Infra-red -absorbing dyes can substantially increase the contrast in deeply located tumor tissues [99]. To test this phenomenon, Dove et al. synthesized plasmonic nanoparticle-templated microbubbles, in which the authors demonstrated dual-contrast enhancement in both ultrasound and PAI by binding avidin-gold nanoparticles to microbubble shells having a biotin component [101]. Indocyanine green (ICG) is an FDA-approved optical contrast agent which can be encapsulated in MNBs for dual mode imaging, protecting it from non-specific molecular interactions and significantly increasing its circulation time [28]. Kim et al. prepared multifunctional microbubbles and nanobubbles for photoacoustic and ultrasound imaging by using polymeric shells of PLGA to encapsulate gelatin-based ink in biological tissues located deeply in phantoms [102]. A gold nanoparticle is an absorbent optical contrast agent that can also be encapsulated in MNBs for PAI, owing to unique chemical and physical properties of gold nanoparticles such as absorption in the NIR range, high photostability and biocompatibility [28]. Dixon et al. synthesized microbubbles with surface-bound gold nanorods, referred to as AuMBs, for ultrasonic and photoacoustic imaging and demonstrated that AuMBs produce significantly higher photoacoustic contrast in deep tissues [25]. McEwan et al. used polymeric microbubbles for sensitizers in sonodynamic therapy [103]. Porphyrin microbubbles have also been used for multimodality imaging [23]. Huynh et al. used ultrasound to burst porphyrin microbubbles into nanoparticles and used them for the multimodal imaging of tumors [104]. These studies concluded that MNBs can be functionalized with photosensitizers for dual modality imaging. 

Photodynamic therapy requires a light source and a photosensitizer that generates a reactive oxygen species (ROS) [6,105,106]. A photosensitizer is a non-toxic drug administered intravenously or topically to a patient [45]. Light of a suitable wavelength is used to activate the photosensitizer, which creates toxic effects via the generation of ROS in the presence of molecular oxygen [107,108]. The phenomenon of dual selectivity based on photoactivation and localization of the photosensitizer makes PDT a favorable therapeutic modality [6]. The efficacy of the treatment is dependent on the tumor localization of the photosensitizer, availability of the oxygen in the target area, the dosage of light, and cellular localization [100,109]. PDT has been limited due to the depth of light penetration through skin and currently various drugs and nanomaterials having higher absorbance in the NIR region are investigated for deep tissue imaging and therapeutics. However, low water solubility and limited tumor accumulation are the drawbacks of such photosensitizers [110]. Suitable carriers can improve the clinical response of such photosensitizers. Suitability of the drug carrier for PDT requires increased structure stability, long circulation life, optimal size to utilize EPR effect, high drug-loading capacity, effective protection against hydrolysis and enzymes, and targeting ability for specific tumors [6,108]. Photosensitizers with better photophysical and photochemical properties can be encapsulated in or conjugated with MNBs which will help in better accumulation in tumors and improved clinical outcomes [106,111]. MNBs are usually stabilized by surfactants to avoid coalescence and they have the capability to incorporate hydrophobic molecules in their cores. Hydrophobic photosensitizers have low solubility and they tend to aggregate when injected intravenously and suitable carriers such as MNBs would also reduce aggregation of photosensitizers and improve treatment effectiveness [6]. PDT strongly depends on the availability of oxygen in the target area because it requires higher oxygen consumption for ROS generation [6,100]. On the other hand, PDT itself is known to cause hypoxia in a tumor microenvironment by destruction of tumor vasculature [106]. However, a lack of oxygen and hypoxia result in the stabilization of HIF-1α, which can reduce the photosensitivity to PDT [87]. The variability in intra-vascular oxygen partial pressure results in non-homogeneous treatment issues [100]. Since PDT is heavily dependent on oxygen availability for ROS generation and overexpression of HIF-1α has also been demonstrated to decrease PDT effectiveness, it is pertinent that a higher volume of oxygen gas at a target site will improve the efficacy of PDT owing to higher generation of ROS and degradation of HIF-1α. Therefore, incorporation of photosensitizers having absorbance in the NIR region in MNBs containing oxygen seems to have promising research prospects.

**Figure 3:**
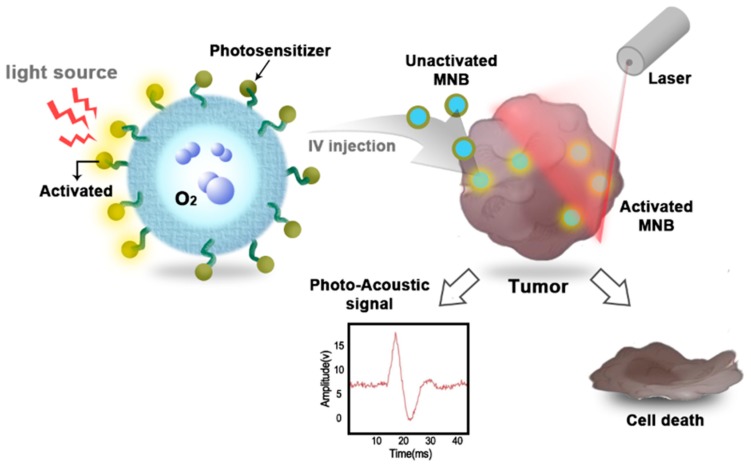
Potential of oxygen-containing MNBs for photoacoustic imaging and photodynamic therapy.

Figure 3 shows an MNB containing oxygen with a photosensitizer drug incorporated in the shell. These MNBs can be used for PAI and PDT, and are more beneficial owing to the oxygen in the core. This yields the benefit of a dual modality imaging technique that shows anatomical, functional, and molecular imaging. Similar MNBs can be used for the enhancement of PDT in the targeted area. As shown in Figure 3, MNBs can be intravenously injected and when laser light is applied, the photosensitizer will be activated. This will improve targeting therapy for tumors and enhance the effectiveness due to the release of the oxygen from MNB simultaneously. More availability of oxygen will help in downregulation of the HIF-1α protein and higher ROS generation. Furthermore, MNBs can be co-administered with other nanocarriers to increase the volume of oxygen in the target area, thereby increasing the efficacy of PDT. MNBs can be applied to multifunctional targeting and they can be tailored to the requirement.

## 7. Conclusions

In this review article, the properties, shell types, synthesis methods, oxygen delivery mechanisms, and potential applications of MNBs in relation with photo-triggered theranostics were summarized. MNBs can be synthesized by various techniques including sonication, emulsification, agitation, and by applying microfluidic devices with different shell and gas compositions to deliver oxygen to a specific site. Phospholipid shells are more flexible, easier to synthesize, and can be functionalized for applications; however, they are limited by their lower half-life. Various techniques of incorporating surfactants like PEG have been used to stabilize phospholipid bubbles at the nanometer-level. Polymeric shells are comparatively thicker than phospholipids and can be tailored to the requirement; however, they have reduced echogenicity and ultrasound is usually required to rupture polymer bubbles for effective gas and drug delivery. However, biocompatible and biodegradable polymers do offer better opportunities for therapeutic purposes. Protein-shelled MNBs also offer stability and biodegradability; however, their diffusion is low. Ultrasound is typically required to cavitate polymer-shelled and protein-shelled MNBs. MNBs can also be tailored for photoacoustic imaging or dual mode imaging by incorporating photosensitizers in the shell or core of the MNBs. 

Hypoxia is a key concern in the treatment efficacy for chemotherapy, radiotherapy, and photodynamic therapy. In this review, we focused on the application of MNBs for increasing the partial pressure of oxygen to reverse hypoxia and hypoxemia, with a potential scope for application in photoacoustic imaging and photodynamic therapy. Oxygen-containing MNBs may enhance the therapeutic efficiency of photodynamic therapy by degrading HIF-1α and increasing the generation of the ROS by simultaneously delivering oxygen and photosensitizers to the targeted tumor. Also, if more clinically suitable photosensitizers having absorbance in the NIR range are incorporated and delivered using MNBs, it might address the skin-penetration problem associated with PDT. Further research is required to evaluate the effectiveness of oxygen carrying MNBs for improving the treatment modalities.

## Figures and Tables

**Table 1:** Characteristics of MNBs used for oxygen delivery by various researchers.

Shell Type	Composition	Oxygen Delivery Method	Synthesis Technique	General Properties	Ref.
Lipid	1,2-Distearoyl-sn-glycero-3-phosphocholine (DSPC), 1,2-distearoyl-sn-glycero-3-phosphoethanolamine-*N*-[amino(polyethylene glycol)] (DSPE-PEG), DSPE-PEG-Biotin (82:9:9)	In vitro cell culture, injection of MNBs in animals	Sonication	Echogenic, biocompatible, easy to synthesize, allows gas diffusion, improved cell penetration owing to endocytosis	[53]
	DSPC, PEG-40-S (9:1)	In vivo injection of MNBs in animals	Sonication		[8,11]
	DSPC + Cholesterol	Injection	High shear mixer		[12,42]
	F-PC, DMPC	Ultrasound exposure	Agitation, sonication		[41]
	DSPC, Poloxamer 188	Injection in deoxyhemoglobin, in vivo animal experiments	Sonication		[10]
	DSPC, DSPE-PEG-2000-Amine, DSPE-PEG-2000-Biotin	Injection in deoxygenated water, in vitro hypoxia reversal in tumor cells	Sonication		[20]
	DSPC, DSPE-PEG-2000 (9:1)	Simulations, injecting oxygen into partially saturated DW, ultrasound targeted release	Sonication		[5]
	DSPC, DSPE-PEG-Folate	Injection into tumors of animal models, ultrasound targeted release	Agitation, mechanical vibration		[91]
	DSPC or 1,2-dipalmitoyl-sn-glycero-3-phosphocholine (DPPC), PEG 40S	Injection of microbubble suspension	Sonication		[39]
	DSPC, *N*-(Carbamoyl-methoxypolyethylene glycol 5000)-1,2-dipalmitoyl-cephalin sodium (DPPE-MPEG5000) (9:1)	In vitro release of oxygen with and without ultrasound	Mechanical agitation		[16]
Protein	Albumin	Injecting oxygen MNBs into nitrogen-saturated PBS	Sonication	Stable, rigid, biodegradable, biocompatible, low diffusion	[9,40]
Polymer	Dextran		Sonication	Non-toxic, biodegradable, thick shell, echogenic	[9,72]
	poly(lactic-co-glycolic acid) (PLGA), Perfluorooctylbromide (PFOB), Pluronic F-68	Dissolution of the gas core	Emulsification		[14]
	Chitosan	Oxygen delivery in physiological solution, cultured cells, without ultrasound	High shear mixer		[13]
	Cellulose	Oxygen delivery to the cell	Sonication		[26,27]
